# Motor neuron vulnerability and resistance in amyotrophic lateral sclerosis

**DOI:** 10.1007/s00401-017-1708-8

**Published:** 2017-04-13

**Authors:** Jik Nijssen, Laura H. Comley, Eva Hedlund

**Affiliations:** Department of Neuroscience, Karolinska Institutet, Retzius v. 8, 171 77 Stockholm, Sweden

**Keywords:** Oculomotor neuron, Fast and slow motor units, Selective vulnerability, Neurodegeneration, ALS, Neuromuscular junction

## Abstract

In the fatal disease—amyotrophic lateral sclerosis (ALS)—upper (corticospinal) motor neurons (MNs) and lower somatic MNs, which innervate voluntary muscles, degenerate. Importantly, certain lower MN subgroups are relatively resistant to degeneration, even though pathogenic proteins are typically ubiquitously expressed. Ocular MNs (OMNs), including the oculomotor, trochlear and abducens nuclei (CNIII, IV and VI), which regulate eye movement, persist throughout the disease. Consequently, eye-tracking devices are used to enable paralysed ALS patients (who can no longer speak) to communicate. Additionally, there is a gradient of vulnerability among spinal MNs. Those innervating fast-twitch muscle are most severely affected and degenerate first. MNs innervating slow-twitch muscle can compensate temporarily for the loss of their neighbours by re-innervating denervated muscle until later in disease these too degenerate. The resistant OMNs and the associated extraocular muscles (EOMs) are anatomically and functionally very different from other motor units. The EOMs have a unique set of myosin heavy chains, placing them outside the classical characterization spectrum of all skeletal muscle. Moreover, EOMs have multiple neuromuscular innervation sites per single myofibre. Spinal fast and slow motor units show differences in their dendritic arborisations and the number of myofibres they innervate. These motor units also differ in their functionality and excitability. Identifying the molecular basis of cell-intrinsic pathways that are differentially activated between resistant and vulnerable MNs could reveal mechanisms of selective neuronal resistance, degeneration and regeneration and lead to therapies preventing progressive MN loss in ALS. Illustrating this, overexpression of OMN-enriched genes in spinal MNs, as well as suppression of fast spinal MN-enriched genes can increase the lifespan of ALS mice. Here, we discuss the pattern of lower MN degeneration in ALS and review the current literature on OMN resistance in ALS and differential spinal MN vulnerability. We also reflect upon the non-cell autonomous components that are involved in lower MN degeneration in ALS.

## Introduction

Initially described by Jean-Martin Charcot in 1869, amyotrophic lateral sclerosis (ALS) is a neurodegenerative disease targeting the motor neuron (MN) system required for voluntary movement. MNs of the autonomic system are less affected [141]. Progressive degeneration of a large proportion of upper (corticospinal) and lower somatic MNs leads to spasticity, muscle atrophy and resulting weakness of skeletal muscles. The first symptoms typically arise in one or more limbs (spinal onset). About 20% of cases present with a bulbar onset, where speech and swallowing problems are noticed first. Whereas spinal onset ALS is fatal at 3–5 years post-diagnosis, bulbar onset ALS patients face a worse prognosis with an average survival of only 2 years. Paralysis of respiratory muscles and subsequent respiratory dysfunction is the cause of death. In a small minority of patients (3–5%) onset of disease occurs in respiratory muscles. This respiratory onset form of ALS has an even shorter prognosis with an average life expectancy of only 1.4 years [171].

The incidence of ALS in Europe is 1–2.5 cases per 100,000 person years, being more common in men than in women at a ratio of 1.3. The point prevalence is approximately 4–6 cases per 100,000 people [1]. The majority of cases occurs without a clearly identifiable hereditary or environmental cause and is identified as sporadic ALS (sALS). Approximately 10% of cases demonstrate direct inheritance (familial ALS; fALS). The first gene discovered to harbour mutations causing fALS was the Cu/Zn superoxide dismutase 1 (*SOD1*) gene [153]. It is estimated that mutations in this gene are responsible for between 10 and 25% of fALS cases and 1–2.5% of all ALS cases. These mutations are thought to induce a toxic gain-of-function of the protein, which becomes prone to misfolding and subsequent aggregation. Subsequently, mutations in several genes have been discovered to underlie ALS, with the most common being TAR DNA binding protein (TARDBP), Fused in Sarcoma (FUS) and Chromosome 9 open reading frame 72 (C9ORF72) [38, 101, 136, 148, 183]. TDP-43, the protein product of TARDBP, and FUS are known RNA-binding proteins with several functions in processing and maturation of RNAs [102]. For TDP-43, a loss of nuclear function due to mislocalisation to the cytosol is thought to trigger ALS [187]. For FUS, mislocalisation also occurs, but here a toxic gain-of-function in either cytoplasm or nucleus appears more likely as pathology still occurs regardless of the presence of normal FUS in the nucleus [128, 164, 170].

The function of the C9ORF72 protein has not yet been elucidated. A massive hexanucleotide repeat expansion in this gene was found to underlie both fALS and sALS cases [38, 148]. It is currently the most common mutation identified in sALS patients. A loss-of-function pathology was initially proposed as the massive expansion was thought to disrupt the normal function of the protein. However, mice lacking the C9ORF72 homologue do not develop a motor-related phenotype [7, 169]. A gain-of-function is, therefore, more likely. It is debated if pathology arises from a gain-of-function at the RNA or protein level or both. RNA–protein aggregates called RNA foci were detected, as well as protein products arising through repeat-associated non-ATG dependent translation (reviewed in [15, 60, 190]).

Genes found to be mutated in ALS are ubiquitously expressed throughout the nervous system as well as in various other tissues, but selectively cause degeneration of somatic MNs. It is currently unclear why these MNs are so vulnerable to mutations in SOD1, FUS, TDP-43 and C9ORF72.

**Fig. 1:**
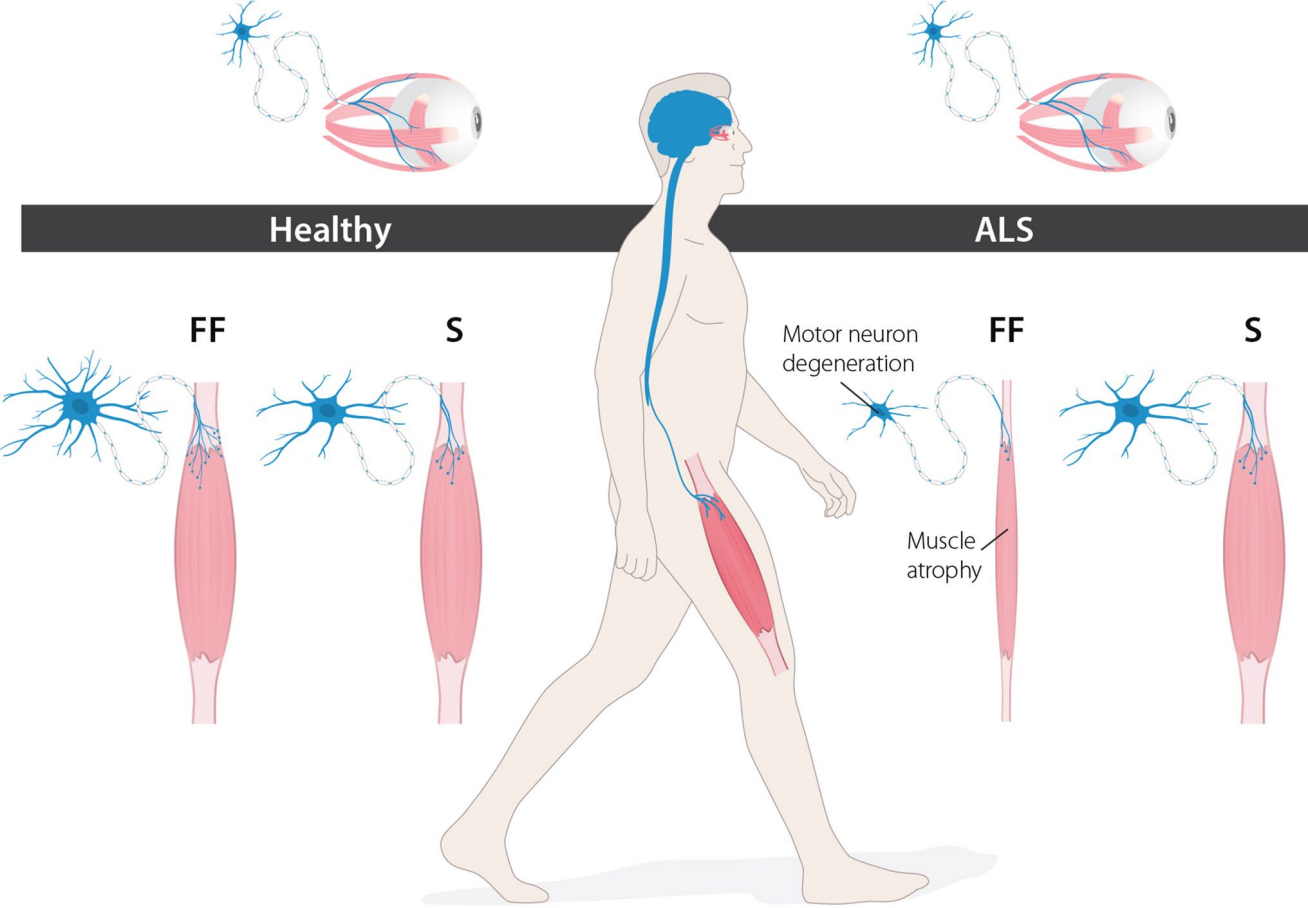
Levels of selective vulnerability in ALS. ALS selectively affects the somatic motor neuron system. Specific motor neuron pools are more resistant, such as the oculomotor neurons, which persist until end-stage of disease. Additionally, subtypes of affected spinal motor neurons also display a gradient of vulnerability. Fast-twitch fast-fatigable (FF) motor neurons degenerate before slow (S) motor neurons

Furthermore, while upper and lower somatic MNs are selectively targeted in ALS, some lower MN subgroups are relatively resistant to degeneration [34, 73]. Selective vulnerability occurs at different levels in the motor system (Fig. 1). Neurons of the oculomotor (CNIII), trochlear (CNIV) and abducens (CNVI) nuclei, which are located in the midbrain and control eye movement, show marked resistance to degeneration in ALS. This allows patients even in late stages of disease to communicate using eye movements, often computer-aided [28, 98]. Additionally, MNs innervating pelvic floor muscles remain relatively unaffected, generally preventing incontinence in ALS patients [30]. Furthermore, there is a gradient of vulnerability among spinal MNs where faster motor units become affected before slower types [147]. Consequently, ‘fast’ muscles relying mainly on glycolysis become paralysed before slow types with a more oxidative metabolism [74].

The reasons for the differential vulnerability among MNs remain largely unknown. Experiments conducted on fALS animal models where mutant SOD1 or TDP-43 have been selectively removed from specific cell types indicate that factors intrinsic to MNs are crucial for initiation of degeneration and early disease [16, 82, 84, 90]. In mutant SOD1 models oligodendrocytes also appear important for initiation of disease [89], while inflammatory events elicited by astrocytes and microglia appear to drive disease progression [16, 54, 194]. Based on the importance of factors intrinsic to MNs for disease initiation, we believe that an analysis of the intrinsic properties of MNs displaying differential susceptibility to disease could reveal mechanisms of neuronal resistance and vulnerability. This in turn could lead to future therapies to prevent the progressive MN loss in ALS.

Here, we aim to review the literature regarding motor neuron degeneration in ALS. We will discuss the selective vulnerability between lower MN subgroups, focussing on the oculomotor system and the gradient of degeneration in the spinal cord. We also reflect upon the involvement of non-cell autonomous events in MN degeneration in ALS.

## Motor neuron degeneration in ALS

Of clear interest to the field of ALS therapeutics is the identification of the primary site of visible pathology within vulnerable MNs, which consist of three clearly defined subcompartments—soma, axon and synapse. A therapy targeting a secondary site of pathogenesis may be too late to have any positive effect on disease outcome.

Careful studies using the SOD1^G93A^ mouse model have investigated the time course of distal and proximal events within MNs [52, 111, 185]. Different genetic backgrounds are known to change the timeline of pathology in SOD1^G93A^ mice, but the pattern of dying-back pathology holds true. One study (where background was not specified) found denervation of NMJs in hind limb muscle at postnatal day (P)47, well before the loss of MN somas at P100 and a decrease in rotarod performance from P78 [52]. A similar pattern was also reported by others (in SOD1^G93A^ mice on a mixed B6SJL and C57Bl/6 background) with a decrease in rotarod performance at P60 preceding the loss of MN somas that occurred after P90 [111]. Also in B6SJL (high expressor) mice denervation at P30 preceded MN loss at P60 [185]. A similar dying-back pattern beginning at neuromuscular synapses was reported also in motoneuron degeneration (Mnd) and progressive motoneuronopathy (pmn) mice, indicating that the NMJ is vulnerable early across diseases with distinct underlying pathological processes [55].

Dying-back pathology is also seen in ALS patients, as initially reported by histological analysis of a patient who died unexpectedly prior to end-stage. Muscle atrophy and denervation were evident in skeletal muscles of the legs and thorax, but no axonal degeneration was observed within the ventral roots and normal numbers of MNs were present within the spinal cord [52]. In ALS patients, electrophysiology has been used to demonstrate early transmission changes in several different muscles. It has been reported that patients have decreased amplitudes of miniature endplate potentials (small depolarisations of the postsynaptic terminal caused by the spontaneous release of a single vesicle of acetylcholine) [119] and a decrease in twitch force of muscle units [41]. Recently, morphological analysis of NMJs was correlated with clinical and electrophysiological data from nine ALS patients, of whom five were early-stage patients and four were long-term survivors of the disease. Morphological abnormalities were found in the incoming nerve, the muscle and the terminal Schwann cells in both early-stage patients and long-term survivors, suggesting again that alterations at the NMJ are present early on in the disease [20].

In line with these results it has been shown that preserving MN somas alone is not enough to rescue mutant SOD1 mice because the axons cannot reconnect at the NMJ once they have retracted enough [42, 62, 155]. This supports the notion that therapy should be aimed at both the soma and the NMJ to optimise the beneficial effect.

Furthermore, in the SOD1^G93A^ mouse upper MNs also appear vulnerable in a dying-back fashion [138]. However, an alternate degeneration pattern can be triggered in these neurons. For example, the loss of ubiquitin C-terminal hydrolase-L1 (UCHL1) caused progressive dying-forward of corticospinal MNs, affecting the cell body first and progressing towards the spinal cord. Meanwhile, interestingly, spinal MNs displayed strong dying-back pathology, with pronounced NMJ denervation but leaving spinal MN numbers unaffected [58]. This indicates that the mechanisms responsible for degeneration and coping mechanisms of upper and lower MNs cope appear distinct.

## The oculomotor system

In mammals, there are six extraocular muscles (EOMs) controlling eye movement. Of these, four are innervated by MNs in the oculomotor nucleus (superior rectus, medial rectus, inferior rectus and inferior oblique). Of the remaining two, the superior oblique is innervated by neurons from the trochlear nucleus, while the lateral rectus is innervated by the abducens nerve.

The EOMs are unique in a number of respects, confounding the task of identifying the key differences responsible for their protection from degeneration in MN diseases (see Box 1).

### Anatomy and innervation pattern of the oculomotor system

**Fig. 2:**
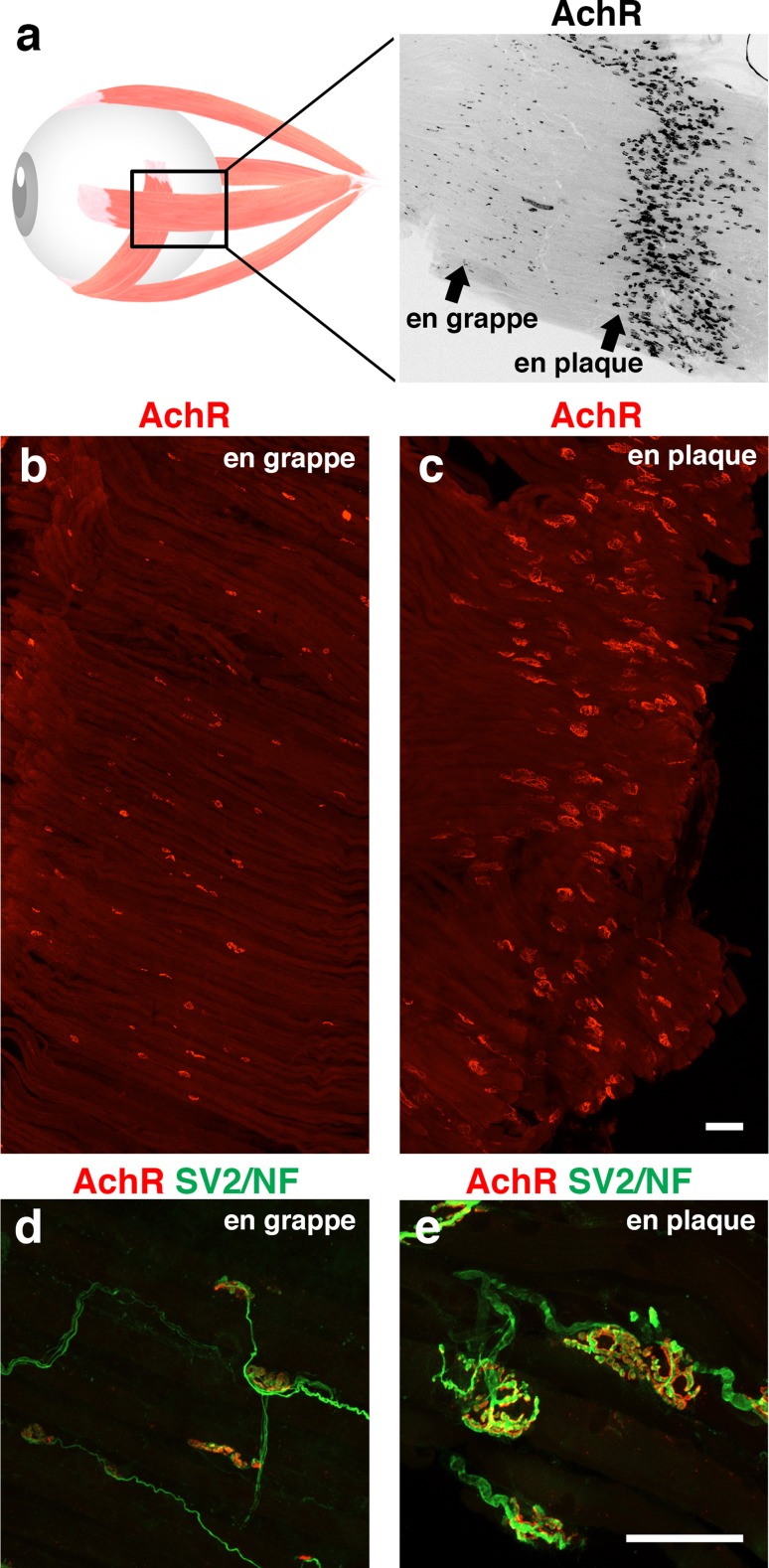
Neuromuscular endplates in extraocular muscle. All six extraocular muscles in mammals have a unique arrangement of neuromuscular endplates. Two distinct bands (rather than one) can be observed (**a**). Extraocular muscles contain a unique band of small *en grappe* endplates (**b**, **d**), besides the regular band of normal-sized *en plaque* endplates (**c**, **e**). Multiple innervation occurs between bands, such that one muscle fibre can have multiple NMJs, one within each endplate band. *Scale bars* 50 μm, *bars* in **c** and **e** also apply also to **b** and **d**, respectively. *AchR* acetylcholine-receptor, *SV2/NF* synaptic vesicle protein 2 and neurofilament 165 kDa

First, EOMs have a different pattern of innervation compared to almost all skeletal muscles. The majority of skeletal muscles have a single point of contact between each muscle fibre and an incoming axon at the NMJ. These NMJs are usually located towards the centre of the fibre, giving rise to the so-called *en plaque* endplates. While 80% of EOM fibres conform to this pattern of singly innervated fibres (SIFs) the remainder consists of multiply-innervated fibres (MIFs). Multiple innervation is achieved with smaller endplates compared to the *en plaque* type, which are organised in grape-like structures, leading to the name *en grappe* endplates. These endplates are located in distinct, more distal bands of the muscle, spatially separated from the regular *en plaque* SIF endplates on the muscle belly region (Fig. 2). In addition to an *en plaque* endplate, multiple *en grappe* endings can be present on the same myofibre, interconnected by the same axon [199]. The MIFs do not respond with the typical ‘all-or-nothing’ rapid twitch response but instead allow for small graded contractions [142, 167]. These contractions remain local and are not propagated along the muscle fibre. It was suggested that these localized, slow contractions finely modulate eye movement or dampen the strong twitch contractions for more stable vision [85]. They also incorporate sensory functions, allowing for reflexive eye movements such as gaze fixation [26, 199]. The somas of both SIF- and MIF-innervating neurons are located in the oculomotor nucleus in the midbrain. MIF-innervating neurons are present in the periphery of this nucleus, while SIF-innervating neurons constitute the centre [27]. Moreover, these neurons receive different projections, likely related to the differential function of SIF and MIF fibres in ocular movement and reflexes [189].

Secondly, EOMs have a distinct composition of ten myosin heavy chain fibre types and uniquely express multiple types within a single myofibre [198]. This contrasts with skeletal muscle, which generally expresses a single isoform of myosin per fibre that is suited to the demands of that muscle. The range of myosin isoforms present in EOMs also includes embryonic and neonatal forms, which are only partly downregulated in adulthood [19], and α-cardiac myosin, more typically found in smooth muscle of the heart [157] (see Box 1). As these isoforms contract relatively slowly compared to the other isoforms, they dampen MIF contraction [85].

EOMs also have a different immunological status, as they contain more negative regulators of the complement pathway. This is likely what renders the EOMs more vulnerable than skeletal muscles in the autoimmune neuromuscular disease myasthenia gravis [143].

**Fig. 3:**
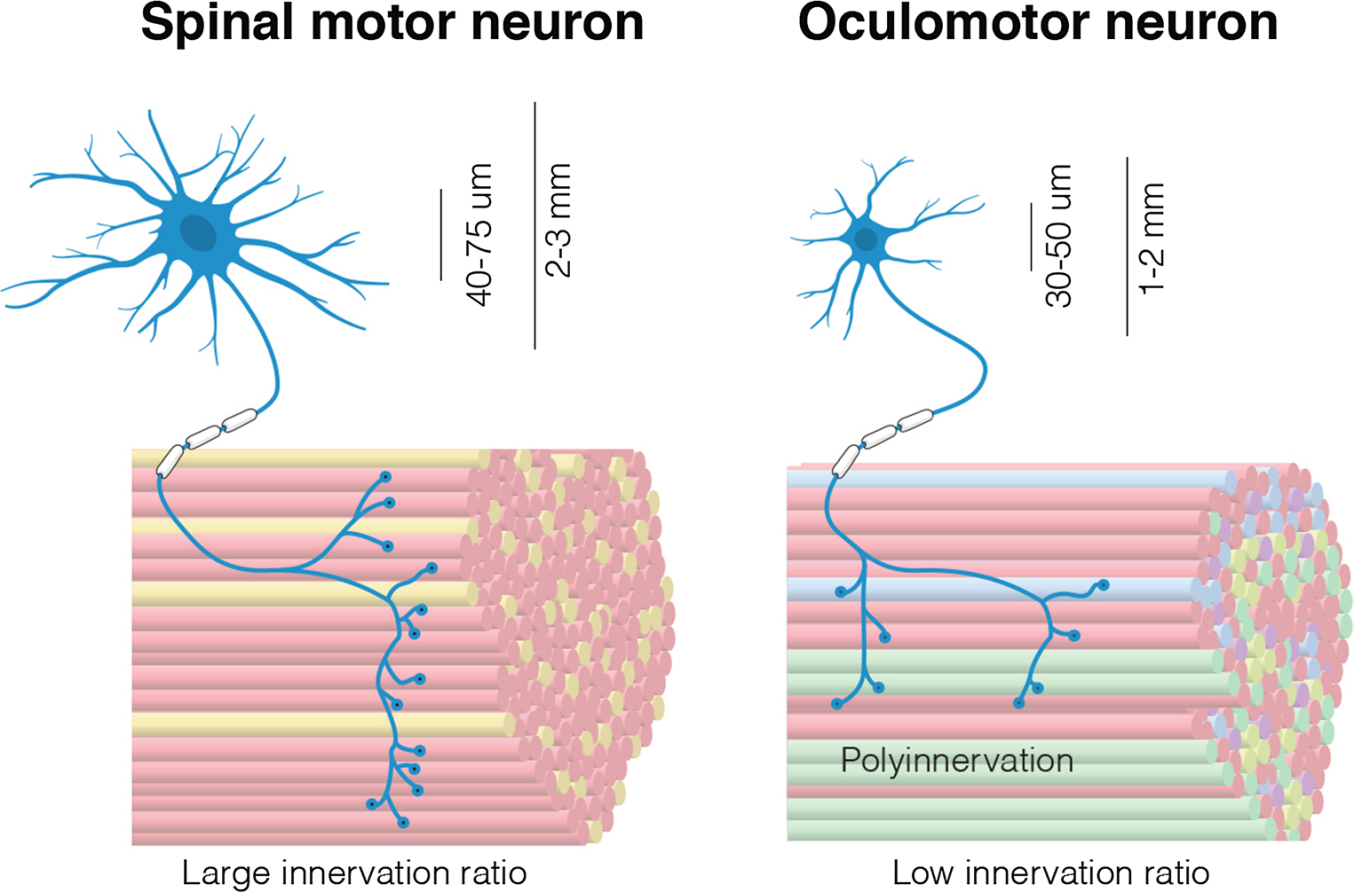
Comparison of a spinal motor unit and an oculomotor unit. Ocular motor units differ from spinal motor units in several key aspects. Oculomotor neurons have smaller somas and their dendritic tree is not as complex as that of a spinal motor neuron. Oculomotor neurons innervate multiple muscle fibre types, whereas spinal motor neurons are generally restricted to the innervation of a single type of muscle fibre. Extraocular muscle itself is more complex, containing many different fibre types, not only those containing the classical skeletal myosins. Oculomotor neurons innervate very few endplates compared to spinal motor neurons, but poly-innervation occurs on a large scale

Motor unit size is small in EOMs compared to other muscles (Fig. 3). Regular fast-twitch skeletal muscles have innervation ratios that often exceed 1:300; each single MN innervates at least 300 muscle fibres. This number can rise to 2,000 in large muscles such as the medial gastrocnemius [23, 25]. In EOMs, the innervation ratio is substantially lower, with ratios as low as 1:5 having been reported [48, 66] (see Box 2). The small motor unit size combined with the presence of multiple innervations likely allows for highly precise regulation of EOM tension and contraction.

**Figure:**
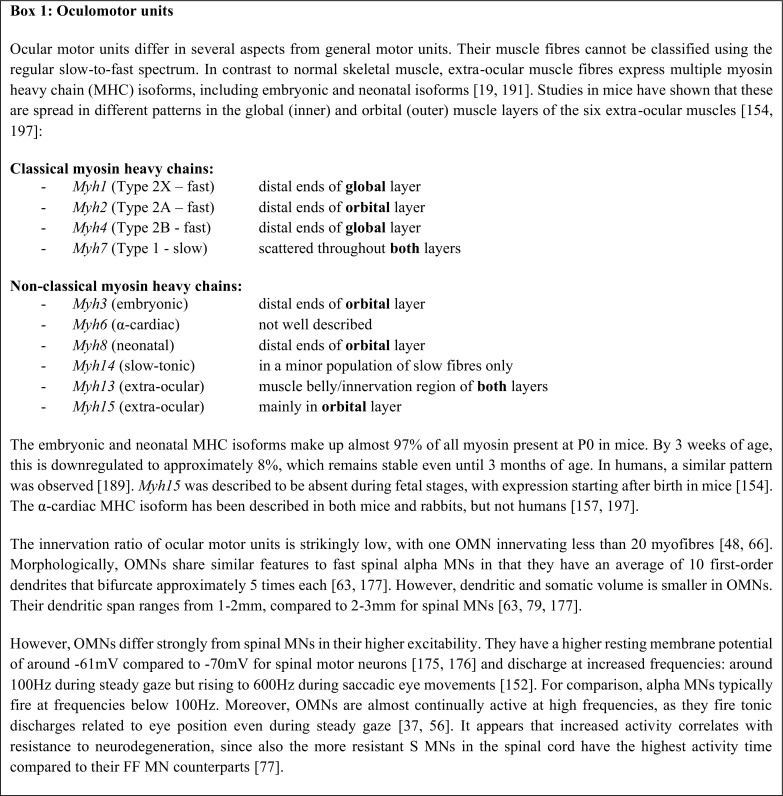

### Embryonic development of oculomotor neurons

OMNs are generated in the ventral midbrain along with dopamine neurons, red nucleus neurons and GABAergic interneurons. These neuron types are specified by the signalling molecules Shh (sonic hedgehog), that is secreted from the floor plate and the notochord, Wnts (wingless-related MMTV integration site) and Fgf8 (fibroblast growth factor 8) that are secreted by and around the isthmic organiser [86, 121, 140, 144, 195]. These morphogens in turn regulate transcription factors that influence differentiation of ventral midbrain neurons [6, 40, 50, 71, 139, 146]. Specifically, OMNs are specified by the transcription factors Phox2a, Phox2b and Lmx1b. Phox2a is expressed already at embryonic day (E) 9 in the forming oculomotor and trochlear nuclei and present in both neuroepithelial precursors and differentiated neurons. Phox2b is expressed around E10.5 and then only in differentiating neurons [139]. Lmx1b is required for the expression of Phox2a [40]. Phox2a in turn is required to drive OMN fate as demonstrated by the lack of both oculomotor and trochlear nuclei in Phox2a knockout mice [139]. Overexpression studies in the chick have demonstrated that Phox2a is also sufficient to generate a complete oculomotor complex consisting of somatic OMNs and visceral Edinger–Westphal neurons [71, 139]. Phox2b on the other hand is sufficient to induce ectopic generation of OMNs in the spinal cord [47, 139], but it is not required for induction of OMN fate in the midbrain [47, 139]. In vitro studies have confirmed that overexpression of either Phox2a or Phox2b in neural progenitors exposed to Shh and Fgf8 can promote a midbrain/hindbrain MN fate [131]. Proper migration and axonal outgrowth of newly born OMNs appears regulated by the Shh-inducible transcription factor Nkx6.1 through the modulation of a number of migration/guidance cues including Unc5c, Robo1 and Slit2 [145]. OMNs express the LIM homeodomain transcription factors Islet-1 and 2 (Isl1/2), but lack expression of the transcription factor Hb9, which defines other somatic MNs. The transcriptome and proteome of OMNs is consequently distinct from other somatic MNs [4, 18, 33, 73].

### Selective resistance of the oculomotor system in ALS

Given the plethora of unique characteristics of the ocular motor system **(**Fig. 3 **)** it is perhaps unsurprising that it responds differently in ALS compared to other motor units—remaining resistant to degeneration even at later stages of the disease. A study of 34 motor neuron disease (MND) patients reported normal ocular motor function compared to control subjects in all patients, except in the presence of Parkinsonism [61]. Clinically, this allows patients in later stages of different MNDs to use eye movement as a communication tool [28, 98]. A post-mortem histological study revealed that the oculomotor nucleus was ‘altered’ in only 4 out of 50 patients studied [105]. No correlation between genetic background and the resistance of oculomotor neurons has been described, so it appears to be a consistent feature of ALS. Additionally, oculomotor resistance occurs across motor neuron diseases. In spinal muscular atrophy (SMA) patients ocular tracking devices are used in later stages of the disease to enable communication [98], indicating the resistance of OMNs. Furthermore, in a mouse model of SMA no denervation was observed in EOMs [34].

OMN resistance has also been reported in multiple mouse models of ALS. In the SOD1^G93A^ mouse, EOMs remain fully innervated at stages where limb muscles show profound denervation [34, 174, 179]. MN counts in brainstem nuclei of end-stage SOD1^G86R^ mice revealed a 48% loss of MNs in the vulnerable facial nucleus, but only a 6% loss in the oculomotor nucleus, which was largely due to one severely affected animal in the sample group [137]. An inducible mouse model of mutant TDP-43 also displayed a similar pattern, with sparing of MNs in oculomotor, facial and trigeminal motor nuclei, but loss of hypoglossal and spinal MNs [168]. In patients with intraneuronal pTDP-43 inclusions in the spinal cord the OMNs rarely contain similar inclusions. In the few cases where such inclusions were found patients had been maintained on respiratory support or had very extensive pTDP-43 inclusion pathology that did not confine to predefined pathology staging [17, 129]. Analysis in end-stage SOD1^G93A^ mice demonstrated that OMNs appeared largely devoid of SOD1 and p62-positive inclusions, indicating that autophagy was unaffected here. Areas surrounding the oculomotor nucleus displayed some astro- and microgliosis at end-stage of disease in SOD1^G93A^ mice, at levels similar to the vulnerable hypoglossal nucleus [5]. As both the vulnerable facial nucleus and the resistant red nucleus showed several fold higher levels of gliosis at this point, it is evident that a time course for glial activation in the brain stem is needed to conclude when and where activation occurs and if this is important for the differential MN vulnerability seen in the brain stem.

### Cell-intrinsic determinants of oculomotor neuron resistance

The selective resistance of OMNs could offer potential clues to mechanisms underlying degeneration in ALS and define the molecular signature that renders a specific subset of neurons more resistant while others degenerate in the face of the same insult. To pinpoint these differences, we previously compared the global transcriptional profiles of OMNs, hypoglossal MNs and spinal MNs isolated by laser capture microdissection from 8-week-old normal Sprague–Dawley rats. This comparison of vulnerable and resistant MNs revealed enriched transcripts for each population [73]. At the protein level, we subsequently showed that OMNs, hypoglossal MNs and spinal MNs have distinct expression signatures [33]. GABA_A_ receptor α1 (Gabra1), parvalbumin, guanylate cyclase soluble subunit alpha-3 (Gucy1a3) and insulin-like growth factor 2 (IGF-2) were persistently expressed in OMNs in mouse and man as confirmed also by immunohistochemistry [4, 33, 73]. Parvalbumin has previously been shown to be protective in ALS [180], but cannot fully explain differential MN vulnerability as it is also highly expressed in vulnerable spinal MNs. There is also great variability in the parvalbumin protein levels among OMNs, arguing against it being a main effector of neuronal resistance [33]. Differences in inhibitory synaptic transmission, mediated by glycine and GABA neurotransmission, could in part underlie the differential vulnerability of oculomotor, hypoglossal and spinal MNs. We and others have shown that Gabra1 is preferentially expressed in OMNs in rodent and control patient tissues [18, 33, 114]. We also demonstrated that Gabra1 remains preferential to OMNs in end-stage ALS patients indicating that Gabra1 could be a candidate for MN resistance [33]. Particularly compelling was the finding of a preferential presence of IGF-2 in OMNs in mouse and man in control and ALS [4, 73]. IGFs are known MN survival factors [49, 165] and viral delivery of IGF-1 to SOD1^G93A^ mice is protective [91]. We, therefore, inferred that IGF-2 could play a role in oculomotor resistance in ALS. This was further supported by the selective expression of the IGF-1 receptor, which mediates survival upon IGF-1/2 binding, on OMNs and extraocular muscles [4]. We also demonstrated that IGF-2 was protective across MNDs, improving survival of induced pluripotent stem cell-derived spinal MNs from both ALS and SMA patients. Viral delivery of IGF-2 to MNs of SOD1^G93A^ mice extended their lifespan with 10%, preserved MN somas and induced axonal regeneration [4].

A microarray analysis of OMNs and MNs of the Onuf's nucleus (both resistant to degeneration in ALS) and spinal MNs isolated from P7 mouse tissues identified 18 genes with more than tenfold difference in expression between the resistant and vulnerable MN groups. Seven of these mRNAs were confirmed using in situ hybridization and showed that Sema3e was enriched in OMNs while Npr3, Egln3, Mmp9, Trhr, Hsd17b2 and Nts were enriched in spinal MNs. It was subsequently demonstrated that ablation or reduction of MMP9 delayed muscle denervation and prolonged survival of transgenic mice. Selective introduction of MMP9 into mice was elegantly shown to be sufficient to induce degeneration of fast MNs, confirming the detrimental role of this metalloproteinase [90].

These studies demonstrate that gene/protein expression analysis of MNs with differential susceptibility to degeneration can be used to identify candidates that protect vulnerable MNs. It also shows that neuronal vulnerability is governed both by a lack of certain intrinsic beneficial factors, as well as the presence of detrimental molecules (see Table 1).

**Table 1:** Key molecules identified as enriched in resistant or vulnerable motor populations and their functional effect upon overexpression or suppression

Oculomotor enriched	Outcome	References
IGF-1	Overexpression protective in vivo, delayed onset and progression and extended lifespan in ALS mice	[91]
IGF-2	Overexpression protective in vitro and in vivo, extended lifespan in ALS mice	[4]
Parvalbumin	Overexpression protective in vivo, delayed onset and extended lifespan in ALS mice	[9]
Glur2	Overexpression protective in vivo, delayed onset and extended lifespan in ALS mice	[173]

Spinal enriched	Outcome	References
Peripherin	Overexpression caused ALS-like disease with MN loss	[8]
Dynein	Mutant dynein (loss-of-function) extended survival of SOD1^G93A^ mice, but not other SOD1 mutant mice	[83]
MMP9	Suppression extended lifespan, overexpression accelerated denervation of FF muscle	[90]

**Figure:**
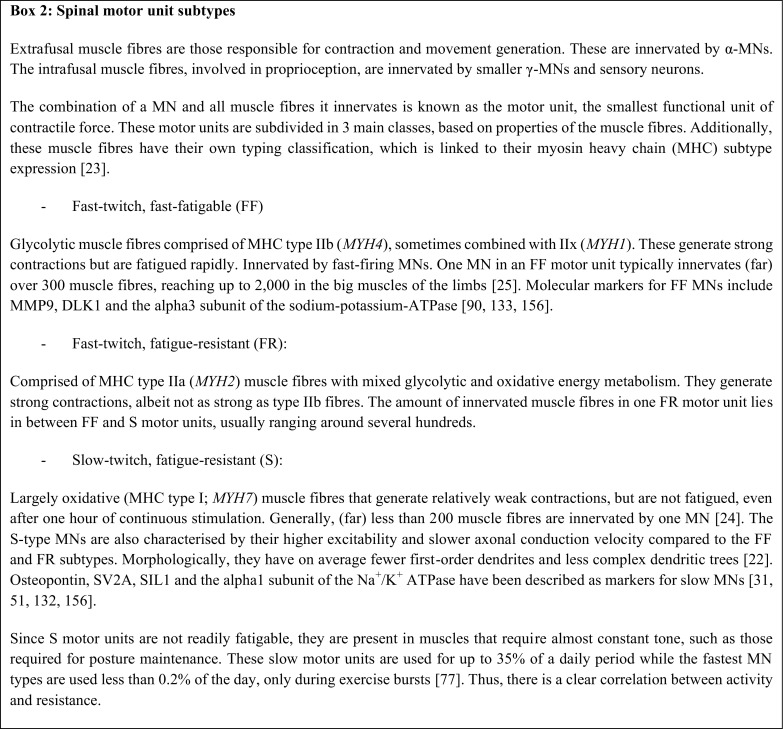

## Differential vulnerability between spinal motor units

Skeletal muscle fibres can be categorised based on their contractile properties. Type I fibres are characterised by mainly oxidative metabolism and a slow-twitch and fatigue-resistant phenotype. Type IIb and IIx muscle fibres use glycolysis as their main energy source and display fast-twitch and fast-fatigable properties. Type IIa fibres form an intermediate class of fast-twitch fibres that retain a level of oxidative capacity.

These subsets of fibres are innervated by distinct α-MN populations, giving rise to three subsets of motor units (see Box 2). Type I fibres form slow (S) motor units, type IIa fibres form fast-twitch fatigue-resistant (FR) motor units and type IIb fibres fast-twitch fast-fatigable (FF) motor units. All fibre types are often present within one muscle, innervated by their corresponding MNs. However, the ratio between slow and fast fibre types varies between muscles, so as to suit each muscle’s function.

In ALS, as demonstrated in the SOD1^G93A^ mouse model, hind limb muscles containing a high percentage (>90%) of fast-twitch type II fibres (medial gastrocnemius, tibialis anterior and extensor digitorum longus) have decreased contractile force before slow-twitch muscles like the soleus are affected. Moreover, motor unit loss was observed at 40 days of age in the fast-twitch muscles. In the soleus muscle, which contains 50% slow-twitch fibres, this loss only became apparent at 90 days of age [74]. This corresponds to other findings showing that MNs innervating type IIb fibres degenerate before type IIa and type I fibres are affected [55, 147, 185].

### Electrophysiological properties of fast and slow motor neurons

The exact aetiology for the selective vulnerability of FF MNs is still unknown, although several hypotheses attempt to explain the difference. The MN subtypes themselves differ first and foremost in their firing rate, hence their naming. The after-hyperpolarization (AHP) latency following an action potential is shorter in FF MNs, allowing for a faster firing rate compared to S MNs [57]. Based on electrophysiological characteristics, lumbar MNs in the SOD1^G85R^ mouse could be clustered into four groups. MN clusters innervating the soleus displayed a slower firing rate compared to those innervating the more fast-twitch tibialis anterior muscle. Motor neuron somas of the fastest firing cluster were hyperpolarized at 2–3 months of age and were subsequently no longer detectable at 4 months of age, at which point the mice had developed motor impairment. Moreover, the number of intracellular aggregations was increased in the MN pool innervating the tibialis anterior [67]. This suggests a disease time course where hyperpolarization of MNs and subsequent intracellular aggregate formation precede muscle denervation and ultimately neuronal cell death, with initiation in the fast firing MNs.

**Fig. 4:**
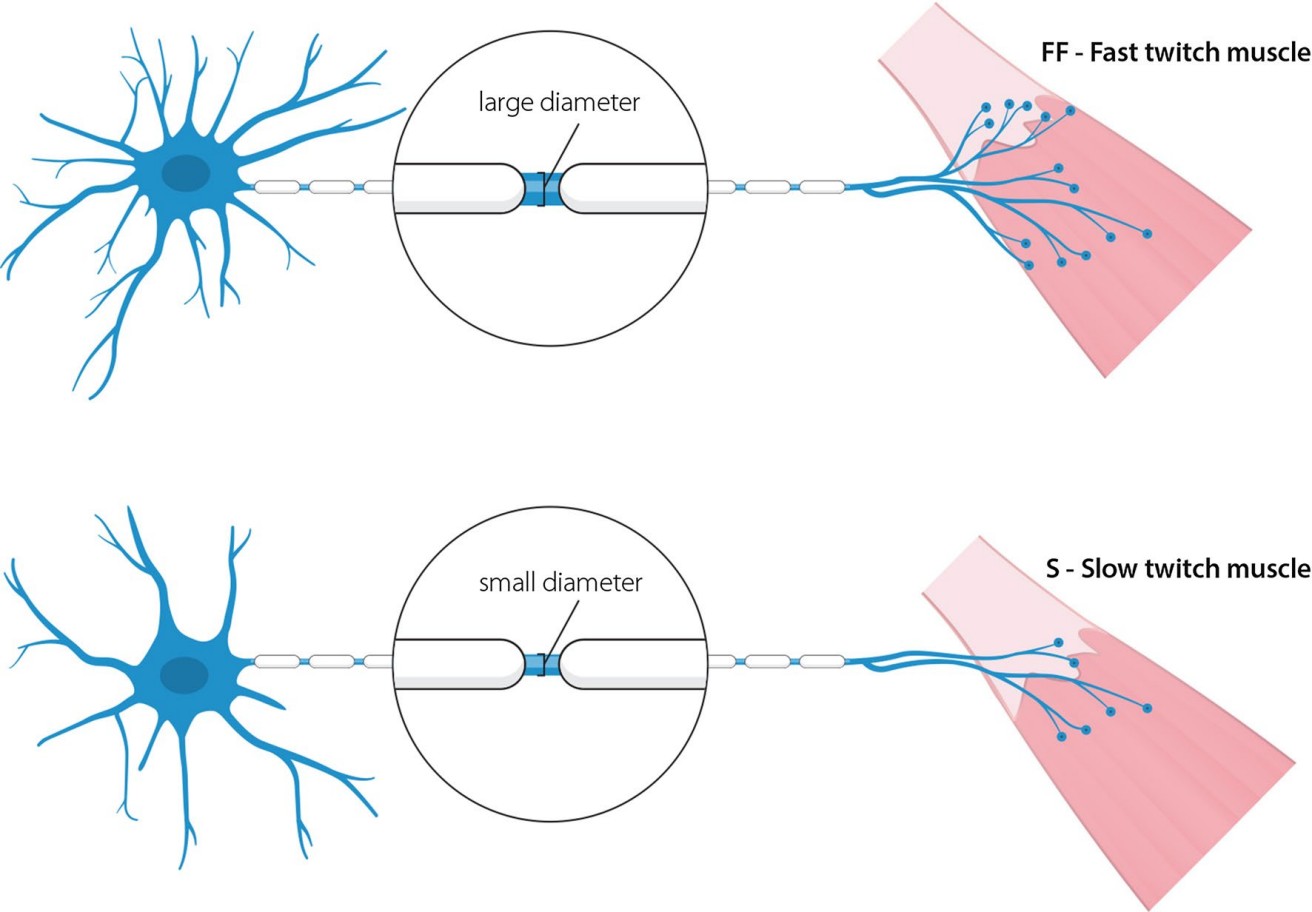
Subtypes of spinal motor neurons. FF (fast-twitch, fast-fatigable) and S (slow-twitch) are the two extremes of spinal motor neurons. FF motor neurons have bigger and more complex dendritic trees and project with larger calibre axons. FF motor neurons innervate a large number of endplates and are thus more involved in high-force movements. S motor neurons are more finely structured and are more suited to their function in slow movements and posture maintenance

Secondly, the input resistance and therefore intrinsic excitability differs between MN subtypes [35]. Highly excitable S MNs are depolarized most rapidly, classically because of their smaller size compared to the larger FF MNs (size principle) [76, 122]. In terms of size, the fast MNs have larger dendritic trees and axon diameters, and therefore, a >20% larger membrane surface compared to S MNs. Within each subpopulation, there is great variability in soma sizes but on average S, FR and FF MN soma sizes are not significantly different (Fig. 4) [22, 35, 93, 185]. Consequently, a recent study in mice could not separate differentially firing MN clusters based on soma size and morphology, again demonstrating that FF and S MN soma sizes are similar [67]. Notably, size scaling alone cannot fully explain the different input resistances postnatally [93], whereas it can in embryonic MNs [118]. This indicates that passive membrane properties differ between FF and S MNs. It has been reported that the remaining spinal MNs in end-stage ALS patients have, on average, smaller soma diameters compared to unaffected individuals [92]. This could be interpreted as a selective resistance by smaller MNs. However, these results could also indicate pathological shrinkage and morphological changes in remaining MNs [95, 96], or be due to the inclusion of small γ MNs in the quantification.

The higher excitability of S MNs has classically been hypothesized to confer resistance to degeneration in the presymptomatic stage of disease. This is consistent with that resistant OMNs are more excitable than both S and FF spinal MNs (see Box 1). Indeed, increasing the excitability of FF MNs presymptomatically by AMPA receptor stimulation and subsequent cation influx reversed the accumulation of misfolded SOD1 protein and protected against cell pathology. On the other hand, partially blocking MN excitability promoted misfolded SOD1 aggregation, although ER stress was reduced, possibly due to reduced intracellular calcium levels [159]. Misfolded SOD1 was later shown to bind to the α3 subunit of the Na^+^/K^+^ ATPase, which was selectively expressed on FF MNs, providing more evidence on the direct interaction between misfolded SOD1 and membrane potential regulation [156].

Exactly why intrinsic hyperexcitability can be protective prior to symptom onset, whereas in later disease stages this process appears to precede and possibly induce MN degeneration is currently unclear. Conflicting reports have further complicated the view on hyperexcitability in relation to degeneration.

Challenging the view that hyperexcitability can be protective in early disease stages, mutant SOD1 mice lacking the ER bound Sigma-1 receptor (S1R) displayed higher excitability and an increased number of action potentials, but also displayed exacerbated disease progression [120]. This implies a modulatory role for S1R in cell excitability and implies that hyperexcitability is certainly not per definition beneficial. It was also proposed that hyperexcitability represents a compensatory mechanism to cope with the disease process itself, which would then be characterised by hypoexcitability. In mouse trigeminal motor units a hyperexcitable shift was observed in the FF MN pool. However, a subset of S MNs actually displayed a hypoexcitable shift [184]. Possibly, FF MNs cope in an aberrant way, since overcompensation towards hyperexcitability induces excessive calcium influx and subsequent ER stress and cell damage.

Hyperexcitability of MNs in ALS has been shown in vitro and in vivo [87, 99, 118, 186]. However, it has been reported that if any shifts are observed at all, they point towards overall hypoexcitability, irrespective of MN subtype [39]. Here, it was speculated that the reason for differential MN vulnerability lies in the ratio of excitatory versus inhibitory synapses on a neuron. As this ratio is larger for fast firing MNs, these could possibly be more sensitive to excitotoxicity. In contrast, another study observed presymptomatic hyperexcitability specific to S MNs [107]. This indicates that there is, as of yet, no consensus view on the presence and role of hyperexcitability in ALS.

### Molecular characteristics of fast and slow motor neurons

On the molecular level, several candidates have been proposed to either promote degeneration of fast MNs or increase resilience in slow MNs. Matrix metalloproteinase-9 (MMP9) was proposed as a vulnerability factor. MMP9 was shown to be expressed selectively in the fast MNs prior to onset of disease in mice, and was suggested to drive ER stress, leading to neuronal degeneration [90]. However, others observed similar MMP9 levels across MN subtypes, based on immunohistochemical observations [67]. On the muscle side, MMP9 expression was shown to be selectively present in ALS-affected muscles, while undetectable in unaffected muscle from ALS patients and controls. However, MMP9 expression was not restricted to a specific fibre type [162]. In ALS patients, MMP9 was observed only in a subset of MNs, although it remains to be demonstrated whether these are FF MNs. MMP9 was also found in serum [12, 109], but could not be detected in CSF [12]. Reduction of MMP9 levels through either pharmacological inhibition or genetic ablation improved lifespan of mutant SOD1 mice [90, 94, 113].

Conversely, the co-chaperone protein SIL1 was shown to be highly expressed in the resistant S MNs, while being progressively reduced during disease in FF MNs. Expression of SIL1 appeared to be modulated by excitation of the neuron. Overexcitation with AMPA increased SIL1 mRNA and protein levels. Moreover, experimental reduction of SIL1 expression in the slow-progressing SOD1^G93A^ mouse model increased ER stress and decreased survival in a dose-dependent manner. Vice versa, overexpression of SIL1 in spinal MNs led to an increase in lifespan [51]. Calreticulin (CRT), another ER chaperone protein, shows a similar subtype-specific pattern of expression. During disease progression CRT levels were reduced solely in the FF MNs, resulting in exacerbation of the *Fas/FasL* (*Fas* ligand) cell death pathway [10]. This MN specific cell death pathway results in ER stress due to nitric oxide generation and caspase-8 activation with subsequent apoptosis. Heterozygous loss of CRT in the SOD1^G93A^ mouse resulted in accelerated denervation of specifically fast-twitch muscle fibres in the tibialis anterior during early disease stages (P45-P55). However, no differences in motor behaviour, motor neuron number or lifespan were evident compared to SOD1^G93A^ mice with normal levels of CRT, indicating that CRT has a role in the modulation of the early peak of ER stress [11].

### ER stress and calcium toxicity

As discussed before, there is a tight link between the differentially expressed genes in S and FF MNs and ER stress. Cellular ER stress is a commonly described molecular mechanism in MNs in several mouse models of ALS, as well as in patient tissue [104]. The cellular coping mechanism for ER stress is the unfolded protein response (UPR). The UPR involves sequestering of chaperone proteins, e.g. SIL1 and CRT, to promote correct protein folding and Ca^2+^ balance within the cell and within the ER. If correct folding of a protein cannot be achieved, it is typically ubiquitinated and thereby marked for degradation by the proteasome complex. Aberrant function of the UPR can interfere with mitochondrial function and ultimately lead to cell death. It was demonstrated that gastrocnemius-innervating MNs (presumed FF) display earlier signs of ER stress compared to soleus-innervating MNs (presumed FR and S). However, a sharp increase in ER stress and a fully activated UPR was found only in the presumed FF MNs just prior to their degeneration [158].

A correct intracellular Ca^2+^ level is crucial for MN function. Regulation of the intracellular Ca^2+^ concentration must therefore be tightly controlled. The presence of Ca^2+^ permeable AMPA receptors on MNs, and their lower intrinsic Ca^2+^ buffering capacity provide clues to why ER stress can be so devastating to these cells in particular [65, 181]. Additionally, in spinal MNs, mitochondria play a large role in Ca^2+^ buffering, increasing the risk of metabolic disturbances under excitotoxic circumstances [65]. An estimated 1/3rd of AMPA receptors expressed by spinal MNs lack the edited glutamate receptor subunit 2 (GluR2), making these channels permeable to Ca^2+^ [64]. Strangely, however, GluR2 comprises nearly 2/3rd of all GluR subunits in these neurons, at both transcript and protein level. This indicates that assembly of subunits into functional AMPA receptors occurs in a regulated fashion, since random assembly would predict that < 3% of AMPA receptors lack GluR2 [64]. In light of selective vulnerability, no difference at the GluR2 transcript level was observed between resistant OMNs and vulnerable hypoglossal neurons in healthy rats [103]. However, in post-mortem human tissue, GLUR2 was enriched in OMNs compared to spinal MNs [18]. While in ALS total GluR2 transcript levels in the spinal cord did not change, posttranscriptional editing failed in almost half of all transcripts, leading to an increase in Ca^2+^ permeable channels [100]. Changes in GluR2 levels in OMNs during disease progression were not investigated. The decrease of properly edited spinal GluR2 proteins and the resulting increase in Ca^2+^ permeability accelerated disease progression in transgenic animals with deficits in this GluR2 mRNA editing [78]. As GluR2 editing changes were not investigated in OMNs in disease, it was hypothesized that these neurons might retain resistance against excitotoxicity. Indeed, it has been shown that OMNs display a reduced inward Ca^2+^ current through AMPA receptors, providing protection against excitotoxicity [18].

Moreover, the intrinsic Ca^2+^ buffering capacity of OMNs is higher compared to spinal MNs [181]. Expression of the Ca^2+^ binding protein calbindin-D28k has been observed to be restricted to resistant OMNs in ALS [3]. It has also been implied in selective neuroprotection in Parkinson’s [46] and Alzheimer’s disease [149]. However, parvalbumin, another Ca^2+^ buffering protein, also stated to be selectively expressed in a pattern similar to calbindin-D28k [3] was recently shown to be present in vulnerable spinal MNs as well. Thus, parvalbumin is likely not the sole contributor to selective MN pool resistance [33]. Nonetheless, overexpression of parvalbumin increased the lifespan of ALS mice [9].

## Genetic disease modifiers

The average survival of ALS patients from time of diagnosis is 3–5 years, but there is a large variability in disease duration, ranging from months to decades, where 10% of patients live for 10 years or more. Notably, the onset and survival among affected family members with the same gene mutation is highly variable [53, 97]. Also in animal models of ALS, based on SOD1 mutations, it has become evident that the genetic background influences disease duration [75, 135]. These facts point to additional genetic factors playing an important role in modifying disease onset and progression [2, 75]. Such genetic disease modifiers could act both cell intrinsically in MNs or cell extrinsically in, e.g., astrocytes, microglia, oligodendrocytes, Schwann cells and T lymphocytes, all implicated in disease [151].

### Atf3, EphA4 and the capacity for motor axon regeneration

**Fig. 5:**
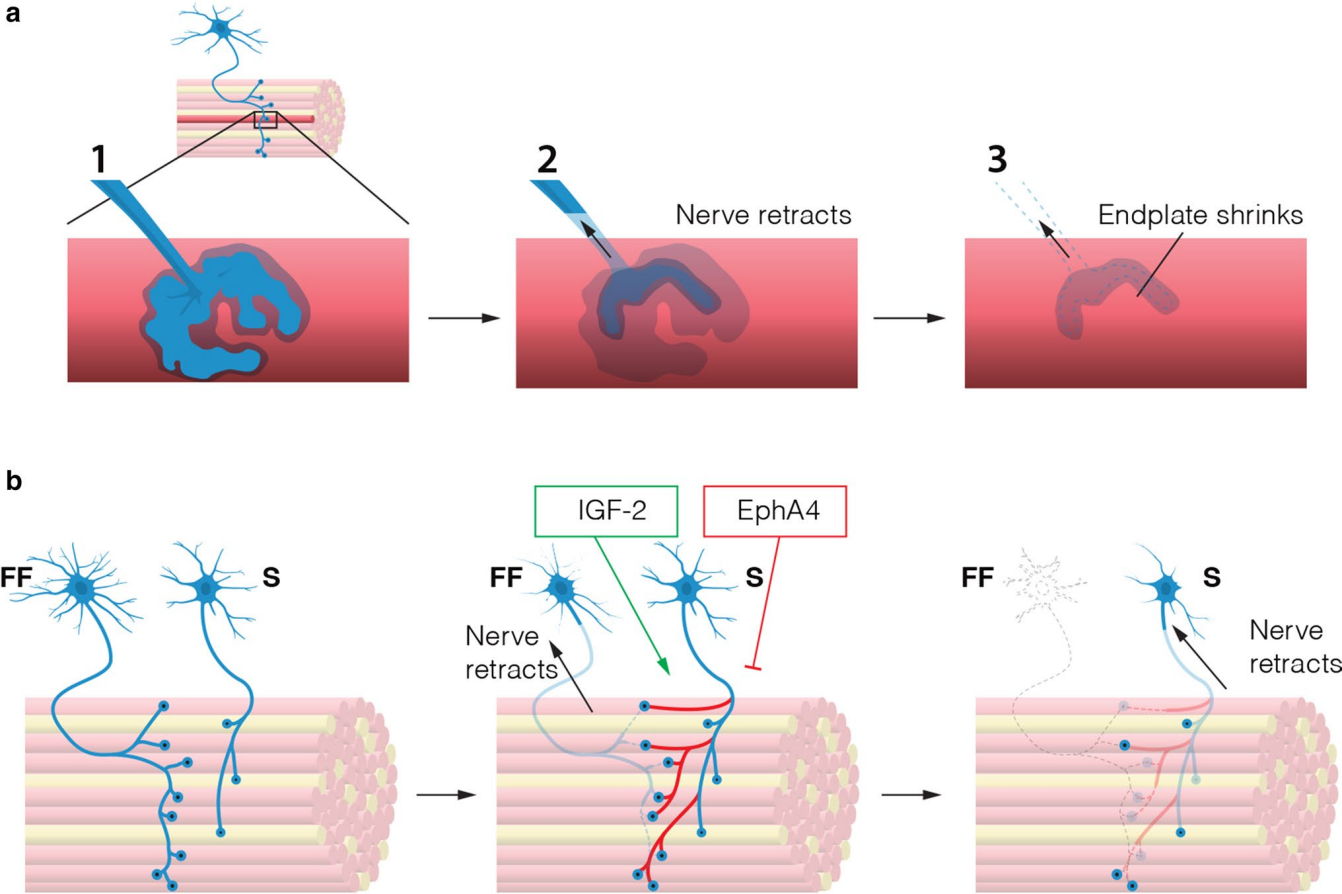
Motor neuron degeneration in ALS. **a** A dying motor neuron retracts its axon from the neuromuscular junction. The axon continues to die back towards the soma. Meanwhile the endplate on the muscle fibre dissolves and shrinks as acetylcholine receptors are internalised and removed. **b** FF motor neurons are the most vulnerable among spinal motors. S motor neurons can temporarily compensate for the loss of FF motor neurons by axonal sprouting and re-innervation of denervated endplates. Overexpression of EphA4 can reduce this effect, while overexpression of IGF-2 stimulates re-innervation, causing ALS mice to live longer. Ultimately, however, also S motor neurons die back and leave the muscle denervated

In ALS, some MNs appear to compensate for the loss of their neighbours and sprout to re-innervate denervated muscle fibres [13, 52, 55, 160]. For example, synaptic sprouting of hypoglossal MNs in SOD1^G93A^ mice was shown to increase the uptake and retrograde transport of viral particles injected into the tongue muscle compared to controls [126]. This correlates with our work showing relatively limited NMJ denervation in the exterior portion of the tongue in the SOD1^G93A^ mouse model [34]. Slow motor units display the highest plasticity and capacity for sprouting [55]. Motor units appear to possess either a degenerative or a regenerative phenotype, with the regenerative motor units initially compensating for NMJ denervation [160] (Fig. 5).

Stimulation of the sprouting capacity of MNs is thought to improve muscle innervation and function of SOD1^G93A^ mice. Hence, overexpression of activating transcription factor 3 (ATF3), which enhances nerve regeneration, led to maintenance of sprouting capacity in SOD1^G93A^ mice up to P120. Disease onset was delayed moderately, but disease progression was not influenced [163]. Although ATF3 overexpression significantly increased muscle innervation at P120, similar to levels in P60 presymptomatic SOD1^G93A^ animals, it did not restore muscle strength to the same extent. This implies that a number of the re-innervated NMJs did not reach complete functionality [163].

Conversely, ephrin receptor A4 (EphA4) expression in MNs impaired NMJ re-innervation (Fig. 5). Blood samples of ALS patients showed an inverse correlation between EPHA4 levels and age of disease onset. To test whether a reduction in EphA4 levels was beneficial for axonal regeneration, SOD1^G93A^ mice were crossed with mice harbouring a heterozygous deletion of the EphA4 gene. These mice showed extended survival by 2 weeks and NMJ innervation was increased at late-symptomatic stages [182]. Interestingly, knockdown of EphA4 also rescued axonopathy induced by mutations in TDP-43, and loss of SMN1, the gene causative for SMA. This indicates cross disease relevance for EphA4 in disease modulation [182].

### PGC-1α and the modulation of muscle fibre type

The peroxisome proliferator-activated receptor gamma coactivator 1-alpha (PGC-1α), a transcriptional coactivator that regulates cellular responses to meet metabolic demands, is a suggested disease modifier in ALS. PGC-1α drives the formation of type I (slow-twitch) muscle fibres which are significantly higher in mitochondrial content and more dependent on oxidative metabolism than type II (fast-twitch) fibres [110]. Transgenic overexpression of PGC-1α generates muscle with increased oxidative capacity and resistance to fatigue and the transgenic mice have improved aerobic performance (the “marathon” mouse) [29]. It is well-established that anterograde signals from MNs to muscle influence muscle specification. Consequently, when a motor nerve that normally innervates a fast muscle is forced to innervate immature muscle destined to become slow, the muscle instead acquires electrical properties of a fast muscle and vice versa [21]. Recently, it was shown that overexpression of PGC-1α in muscle resulted in an increased frequency of MN terminals positive for the synaptic vesicle protein SV2A, a postnatal marker of slow MNs [31]. This indicates that muscle can influence MN innervation either through inducing selective synapse elimination, sprouting or MN identity conversion through retrograde signals [31]. As S MNs are more resistant to degeneration in ALS than FF MNs and as enhanced mitochondrial activity associated with Type I muscle could be beneficial, the effects of PGC-1α overexpression in ALS mice have recently been explored. In one study, where PGC-1α was specifically overexpressed in neurons in SOD1^G93A^ mice, motor performance by rotarod was improved in later stages. Survival was increased by 10 days and MN somas were preserved compared to regular SOD1^G93A^ mice [196]. Another study overexpressed PGC-1α selectively in muscle of SOD1^G37R^ mice, resulting in improved mitochondrial biogenesis and muscle function, as measured by hind limb endurance, treadmill, running wheel and open field. However, there was no effect on disease onset, length or survival and there was no effect on motor axon or NMJ preservation [36]. Collectively, these studies indicate that PGC-1α is beneficial for muscle function in ALS, but that muscle-specific expression is not sufficient to preserve MNs. This also indicates that expression of PGC-1α in muscle likely does not result in a full conversion of vulnerable FF MNs into more resistant S MNs.

## Non-cell autonomous disease processes

Selective ablation of mutant SOD1 in cell types other than MNs in transgenic mSOD1 overexpressing mice has revealed that MN degeneration occurs through an interplay between cell autonomous and non-cell autonomous processes, involving astrocytes, microglia, oligodendrocytes, Schwann cells and T lymphocytes. The initial study, utilizing chimeric mice, showed that MNs surrounded by non-mutant cells survived longer [32], but the specific cell types involved were not identified. Here we give an overview of the cell types at play.

### Astrocytes

Astrocytes are fundamental to synthesis and catabolism of neurotransmitters and amino acids in the central nervous system (CNS). They represent a glycogen reserve and are important in protecting neurons from antioxidants [14, 116]. Astrocytes also control structural and functional plasticity of synapses in the CNS and influence neuronal excitability [166, 178]. However, under influence of activated microglia a subpopulation of astrocytes can become neurotoxic, triggering death of CNS neurons [108]. Removal of mutant SOD1 from astrocytes by crossing loxSOD1^G37R^ mice with GFAP-Cre mice resulted in a delayed microglia response and a 15% increase in lifespan, which was most evident in the late phase of progression. Importantly, removal of mutant SOD1 from astrocytes did not affect disease onset [194]. Astrocytes overexpressing mutant SOD1 appear selectively toxic to MNs in vitro, leaving interneurons unharmed [43, 44]. Notably, not all cells that have high levels of mutant SOD1 are harmful to MNs, as fibroblasts overexpressing mutant SOD1 do not exert toxicity onto ALS MNs [43]. The selective toxicity of astrocytes onto MNs has been used extensively to model ALS-like degeneration in vitro using early postnatal astrocytes [43, 44, 72, 117, 134].

Astrocytes derived from sporadic ALS patients also appear harmful to motor neurons, as shown using astrocytes derived from post-mortem spinal cords [68] and through direct conversion of patient fibroblasts into astrocytes [125].

It is not yet clear if MNs that are relatively resistant to degeneration in ALS are less susceptible to toxic astrocytes or if there is less neuro-inflammation around these resistant cells throughout disease. S and FF spinal MNs are located in close proximity to each other and thus likely have equal exposure to detrimental astrocytes. Therefore, cell-intrinsic differences are more likely the reason for the difference in their demise. It is not known if resistant MNs of Onuf’s nucleus and oculomotor nucleus show differential vulnerability to astrocyte toxicity and/or if the astrocytes surrounding these nuclei are distinct in their response to ALS. Astrocytes within the ventral spinal cord have positionally distinct identities determined by Hox transcription factors [80]. As Hox genes pattern the anterior–posterior axis of the developing embryo, it is likely that rostral astrocytes have identities distinct from caudal astrocytes, just as the MNs they surround do. Therefore, it is possible that astrocytes in the midbrain could show a different response to ALS-causing mutations than spinal cord astrocytes. However, this remains to be investigated. Furthermore, astrocytes in the ventral spinal cord can be distinguished from astrocytes in the dorsal spinal cord through a number of markers, in particular Sema3a, which is required for proper organisation of MN circuits. Loss of Sema3a in these astrocytes leads to selective degeneration of alpha but not gamma MNs [80, 130]. It remains to be investigated if Sema3a expression is altered in astrocytes in ALS and also if Sema3a expression is regulated along the anterior–posterior axis of the CNS.

### Microglia

Microglia are resident immune cells of the brain and spinal cord. They arise from primitive macrophages from the yolk sac and populate the CNS during early development (reviewed in [59]). Microglia are an important component of the inflammatory response to injury and pathogens, and they also influence synapse formation and neurogenesis [161]. Microglia are activated early during disease pathogenesis in ALS [69]. Genetic removal of mutant SOD1 from microglia had no effect on disease initiation or early disease, but significantly slowed later disease progression resulting in an increased mean lifespan of 99 days (a 30% increase in lifespan) [16]. Moreover, adult microglia overexpressing mutant SOD1^G93A^ were toxic to wild-type mESC-derived Hb9-GFP MNs. Notably, neonatal microglia were not found to be toxic to MNs, indicating that the microglia toxicity is acquired and not innate [54]. It remains to be investigated if adult MNs are more susceptible to microglia than the embryonic or early postnatal MNs used in all current and past in vitro studies. This could be technically challenging, but potentially investigated using inducible MNs generated by direct reprogramming of fibroblasts from an aged person, as this procedure should allow generated neurons to retain the age of the donor [123]. Mechanistically, it appears that NF-κB activation within microglia plays a role in MN death. Deletion of NF-κB signalling from microglia extended disease progression in SOD1^G93A^ mice without an effect on disease onset. Consequently, survival was improved by 20 days (a 14% increase) [54]. The authors found no difference in toxicity between brain and spinal cord microglia, implying that these cells lack positional difference in toxicity/activation capacity or that such differences cannot be detected due to the exceptional migratory properties of microglia along the rostro-caudal axis of the animal. Removal of a cysteine/glutamate antiporter, xC, which is enriched on microglia and increased in ALS, decreased glutamate release and the production of nitric oxide, TNF alpha and IL-6 by microglia. Functionally, removal of xC in SOD1^G37R^ mice resulted in an earlier onset of disease but slowed the progression [124]. Thus, xC is a mediator of microglial function which could potentially be modulated after onset of symptoms to prolong lifespan of patients.

### Oligodendrocytes

Oligodendrocytes, which are derived from oligodendrocyte precursor cells (OPCs), myelinate axons in the CNS. They can extend processes to form myelin sheaths around up to 50 neurons and provide crucial metabolic support to these. The monocarboxylate transporter 1 (MCT1 or SLC16A1), which transports lactate, pyruvate and ketone bodies, is highly expressed within oligodendrocytes [150] and down-regulation of this transporter resulted in axon damage and neuronal loss in animal models. Furthermore, ALS patients appeared to show reduced levels of this transporter, indicating the oligodendrocytes could be less metabolically supportive in ALS [106]. Recent findings demonstrate that ALS patients and transgenic ALS mice display demyelination in grey matter regions of motor cortex and spinal cord caused by a progressive degeneration of oligodendrocytes [89]. Simultaneously there is extensive recruitment and proliferation of NG2^+^ OPC in fALS mice [88, 89], similar to that seen in relapse-remitting multiple sclerosis (MS). Nevertheless, while new oligodendrocytes constantly differentiated from OPCs in the fALS mice, they failed to mature. When mutant SOD1 was removed from oligodendrocytes, disease onset was delayed and the survival of fALS mice was prolonged, suggesting that impaired oligodendrocyte function contributes to MN loss in SOD1-related ALS [89]. Whether this holds true for sALS and other forms of fALS remains to be investigated.

### Schwann cells

Schwann cells originate from neural crest cells and are the myelinating glia of the peripheral nervous system. In contrast to oligodendrocytes each Schwann cell myelinates only one axon. Individual Schwann cells myelinate approximately 100 µm of an axon. Thus, a one meter long motor axon can be covered by up to 10,000 individual Schwann cells. During development, Schwann cells are crucial for MN survival. Furthermore, neuron-derived factors guide differentiation and survival of Schwann cells along the axons [193]. In ALS, where motor axons retract and regenerate, fibres in the ventral root show segmental demyelination and remyelination to an extent greater than in control patients, indicating Schwann cell involvement [70]. If interaction between Schwann cells and motor axons could be made more efficient, for example by modulating neuregulin-1 (Nrg1) levels [172], it could be possible to improve regeneration and potentially improve function in ALS. Indeed, viral mediated delivery of Nrg1 into the gastrocnemius muscle of SOD1^G93A^ mice improved local collateral sprouting of motor axons [115].

While genetic reduction of mutant SOD1 from astrocytes, microglia and oligodendrocytes can confer protection to MNs (see sections above), reduction of mutant SOD1^G37R^ within Schwann cells did not affect disease onset, but unexpectedly reduced survival by accelerating disease progression of ALS mice. Here, end-stage was reached on average 42 days earlier, reducing the mean survival by 10% [112]. Mutant SOD1 was decreased both in Schwann cells surrounding peripheral axons and in terminal Schwann cells at the NMJ. The reduction in mutant SOD1 was accompanied by a selective decrease in IGF-1 in the nerves [112]. This reduction in IGF levels would reduce the capacity of MNs to regenerate and form new NMJs with denervated muscle which is a process that prolongs the disease progression phase. This is exemplified by the inverse relationship between EphA4 and duration of disease in ALS patients [182] and with the successful IGF-1/2 treatments in mice [4, 91]. However, the removal of mutant SOD1 from Schwann cells does not, per definition, exacerbate disease. In the SOD1^G85R^ model knock-down of mutant SOD1 in Schwann cells delayed disease onset and extended survival slightly [188]. This SOD1 mutant retains no enzymatic activity, unlike the SOD1^G37R^. Probably the increased dismutase activity in the SOD1^G37R^ mutant partly ameliorates the toxic effects of the mutation in Schwann cells. Conversely, in the SOD1^G85R^ model Schwann cells are only burdened by the mutation, explaining the beneficial effect upon mutant SOD1 knock-down. Combined, these studies highlight a strong dependence of Schwann cells on dismutase activity.

### Muscle

Nerve cells are produced in excess in the developing nervous system and subsequently compete for the limited amount of survival factors that are secreted by their target cells. Only the cells that receive the appropriate type and level of survival signals are maintained. During normal development, MNs in the lateral motor column undergo apoptosis, in which 40% of MNs are lost. Increasing the muscle target area by transplantation of a second leg bud can partially inhibit the MN loss, demonstrating the importance of muscle in the normal survival of MNs [81]. Retraction of the presynaptic motor terminal from muscle endplates is an early event in ALS. Nonetheless, the involvement of muscle in ALS disease onset and progression is highly debated. Several studies have investigated the pathological consequences of modulating the levels of mutant SOD1 in muscle with opposing conclusions drawn from the data received. In one study, mutant SOD1^G93A^ was reduced in MNs by AAV-mediated siRNA delivery to muscle and retrograde transport to MN somas, resulting in functional improvement. In comparison, muscle-restricted reduction of mutant SOD1 to 50% using lentivirus-mediated siRNA, which did not transport retrogradely to MNs, showed no effect [127]. Thus, either muscle is not a primary target in ALS, or reducing mutant SOD1 in this tissue to 50% was not sufficient to reduce toxicity.

Another study addressed the issue of mutant SOD1 action in muscle by selective overexpression of SOD1^G93A^ in muscle. These mice developed progressive muscle atrophy and mitochondrial dysfunction. Interestingly, PGC-1α expression was induced by mutant SOD1 and consequently a switch to more oxidative fibres was seen. Even a mildlevel of overexpression of mutant SOD1 had a negative effect on muscle morphology indicating that the decrease in SOD1 levels in muscle accomplished in the study by Miller and colleagues [127] might not have been sufficient. However, mutant SOD1 levels in muscle alone did not appear sufficient to induce MN degeneration [45]. This would indicate that while mutant SOD1 can induce pathology in muscle, it is not sufficient to drive disease systemically.

A more recent study aimed to analyse the effect of muscle-specific overexpression of both wild-type and mutant SOD1 using the α-actin promoter. Rather surprisingly, MN degeneration was seen with both wild-type and mutant SOD1 overexpression [192]. The detrimental effect observed upon wild-type SOD1 overexpression could perhaps be explained by disruption of endogenous gene expression (unrelated to SOD1) in the transgenic mice. However, this remains to be investigated. The neurodegenerative effect upon mutant SOD1 overexpression is likely explained by the promoter used in the study. While α-actin is thought of as a muscle-specific transcript, in situ hybridization images in Allen Brain Atlas show that MNs also express high levels of α-actin (*ACTA1*), http://mousespinal.brain-map.org/imageseries/show.html?id=100069897. Thus, SOD1 is highly likely to have been overexpressed in multiple cell types even though this was not detected at the time points analysed.

## Conclusion

While somatic MNs are selectively vulnerable in ALS and degenerate in a retrograde fashion, OMNs are resistant to degeneration and stably innervate their muscle targets throughout disease. In the spinal cord relatively resistant S MNs have the capacity to remodel their connectivity with muscle during early disease and partially compensate for the loss of vulnerable FF MNs. Comparative analysis of cell intrinsic properties of these resistant and vulnerable MN groups has begun to reveal the underlying mechanisms of selective vulnerability, resulting in multiple potential targets for future therapies, some of which appear protective across MNDs. Cells other than MNs contribute to disease initiation and progression in ALS and further investigation could reveal if these also contribute to differential vulnerability among MNs.
